# Optimizing the Person-Environment Fit in community childcare and parenting support systems: a pathway to improved wellbeing for children and parents

**DOI:** 10.3389/fpubh.2026.1740064

**Published:** 2026-04-14

**Authors:** Jiehui Geng, Peng Zeng, Jinxuan Li, Ninghan Xu, Chenpeng Wei, Liangwa Cai

**Affiliations:** School of Architecture, Tianjin University, Tianjin, China

**Keywords:** child- and parent- friendly, community childcare and parenting support system, Pearson correlation analysis, Person-Environment Fit Theory, random forest importance analysis

## Abstract

With societal advancement, the increasing burden of childcare and parenting has significantly impacted the quality of life and mental wellbeing of both children and parents, posing serious challenges to family stability and sustainable social development. As the primary living environment for children and parents, communities play a crucial role in meeting their daily needs. However, existing research largely proposes generalized child- and parent-friendly strategies at the urban scale, with limited attention paid to the development of targeted community-level childcare and parenting support environments. Grounded in Person-Environment (P-E) Fit Theory, this research develops an applied analytical framework for a Community Childcare and Parenting Support System (CCPSS). It aims to examine the associations between community environmental subsystems and the satisfaction of multi-level needs among children and parents, in order to enhance their wellbeing. A questionnaire survey was conducted in the communities within Heping District, Tianjin, China, collecting data from both children and parents. Pearson correlation analysis and random forest-based importance ranking were used to examine the associations and relative contributions of different community environmental subsystems to the need satisfaction levels of children and parents. The results indicate a significant positive correlation between the fulfillment of childcare and parenting needs and community environmental systems, with different environmental dimensions contributing to varying degrees. This research extends the application of P-E Fit Theory to the parenting context and provides theoretical and empirical evidence to inform the development of more targeted CCPSS at the community level.

## 1 Introduction

Parents face significant psychological and life pressures in the parenting process, which not only severely affect their physical and mental health but also compromise their quality of life. This, in turn, can reduce their willingness to have children, leading to long-term consequences for population structure and the sustainable development of society (1, 2). Additionally, children, due to their physiological characteristics, are highly dependent on parental accompaniment in daily life. The fulfillment of parenting needs and the alleviation of parenting stress are directly linked to children's quality of life. Therefore, developing a support system that addresses the needs of both children and parents as an integrated whole can effectively improve the quality of life for both groups, reduce parenting stress, and enhance parental attitudes toward childcare, ultimately supporting the sustainable development of social structures.

Many countries have already recognized this issue and have developed support strategies at the family level. For example, the UK's “Sure Start” program aims to provide universal community support services for children under four and their parents, including “health services, early learning and childcare, parenting support, and parental employment support,” to promote family health and development while reducing inequalities in parenting environments (3). The United States' “Head Start” program emphasizes family involvement as a key to the program's success, actively engaging parents and children in early learning and development, health and wellness, family support, and services (4). Japan's “Policies Supporting Children and Child-Rearing” introduces market competition mechanisms that center on the needs of parenting families, with government subsidies provided to promote preschool education and care (5). In 2023, China proposed the establishment of a “universal” family support system to reduce parenting pressure (6). However, these measures mainly focus on policy and economic aspects, offering universal support services at the macro level, with limited exploration of the childcare support needs at the community spatial level.

Communities, as the primary living environment for children and parents, play a critical role in improving their quality of life and alleviating parenting stress (7–9). However, current support systems mainly propose generalized measures based on urban spaces, services, and policies, lacking a systematic and targeted approach, which limits their effectiveness in addressing the complex needs of parents and children (10). Furthermore, research on community support systems primarily has predominantly focused on the provision of child-friendly facilities, with less attention given to parent-friendly strategies (11). Therefore, the construction of a targeted and refined community childcare and parenting support system-based on the matching relationship between children and parents needs and the community environment system, represents a key pathway for addressing current parenting challenges and holds significant practical importance.

This research aims to construct a Community Childcare and Parenting Support System (CCPSS) that aligns actual needs with the community environmental conditions to ensure targeted and refined support measures. By establishing this support system, governments and planners can make more accurate and effective community development decisions, and effectively improve the quality of life and alleviate parenting pressure. First, this study reviews and analyzes the theoretical foundation for constructing a CCPSS, establishing the theoretical framework and methodology for the system. Second, this research focuses on Heping District in Tianjin, China, employing a combination of traditional statistical analysis and machine learning to explore the relationship between the needs of children and parents and the community environment system. The results of the Pearson correlation analysis and random forest importance analysis are then discussed. Finally, based on the findings, recommendations for constructing the CCPSS are proposed, and directions for future research are explored.

## 2 Community childcare and parenting support system theory construction

This study, grounded in Person-Environment (P-E) Fit Theory and incorporating environmental behavioral theory, develops an applied analytical framework for a CCPSS within the context of community-based childcare. By systematically organizing and aligning the need hierarchies of children and parents with community environmental subsystems, the framework provides an operational tool to support community-level childcare service planning and environmental optimization.

### 2.1 Person-Environment Fit Theory

The P-E Fit Theory conceptualizes the interactive relationship between individual needs and the surrounding environmental characteristics, emphasizing that the functionality of the environment should align with individual needs (12). Originating from Parsons' environmental fit model, which focused on career decision-making, this theory posits that individuals exhibit more positive behaviors and psychological wellbeing when their needs are met within an appropriate environment (13). The core relationship within the theory is as follows:


$$\begin{array}{l} B=f\left(P,E\right) \end{array}$$ (1)

Where *B* represents personal life satisfaction, *P* denotes individual needs, and *E* refers to environmental characteristics.

In recent years, the P-E Fit Theory has gained widespread application in studies of wellbeing, underscoring the significant role of fit between personal needs and environmental conditions in influencing happiness and satisfaction. Jokela et al. (14) suggest that individuals residing in communities that match their personality traits tend to experience higher life satisfaction. Kahana et al. (15) developed the congruence model, applying P-E Fit Theory to assess the fit between individual preferences and community environmental characteristics. A higher congruence level indicates greater satisfaction of individual needs, ultimately leading to enhanced residential satisfaction. In summary, the P-E Fit Theory conceptualizes the degree of fit between individual needs and environmental factors. The stronger the environment's ability to meet personal needs, the higher the fit between the individual and the environment, thereby contributing to greater life satisfaction.

The effective functioning of a CCPSS depends on the extent to which the diverse needs of children and parents are aligned with community support environments. Drawing on the P-E Fit Theory, this study develops a research framework centered on needs and environmental subsystems. Through quantitative analysis of their degree of fit, the framework provides an empirical basis for the design and refinement of the CCPSS.

### 2.2 The application and breakdown of Person-Environment Fit Theory in constructing the community childcare and parenting support system

The key to constructing the CCPSS lies in analyzing the fit between the needs of children and parents and the community childcare environment system. Therefore, it is essential to first conduct a systematic classification analysis of the needs of children and parents and the environmental systems within the community.

#### 2.2.1 Analysis of children's and parents' needs

Understanding the individual needs of children and parents in community settings provides a foundation for designing CCPSS. To systematically characterize the need structure of different groups at the community level, this study adopts Maslow's hierarchy of needs, categorizing individual needs into five levels. This hierarchical structure is systematic, progressive, and universally applicable, making it well-suited for analyzing the multidimensional and complexity of individual needs (16, 17).

Based on this theory, the study conducts a layered analysis of the needs that should be addressed within communities, emphasizing not only the fulfillment of fundamental physiological and safety needs but also higher-order needs related to belongingness, esteem, and self-actualization. The specific needs for children and parents were identified through a systematic review of the literature in databases such as Web of Science, focusing on research related to child development needs, child-friendly communities, parenting support, and parental needs. Relevant need elements were synthesized and organized to construct the framework presented in Table 1.

**Table 1:** Needs of children and parents in the community.

Hierarchy of needs	Children	Parents
Physiological need	•Promoting physical activity (63) Physical infrastructure at the child scale (64)	•Community environment for promoting exercise and activity (65) Supporting basic living and caregiving conditions for daily childcare; Access to retail facilities; Access to primary healthcare services; A safe and stable residential environment (64)
Safety need	•Safety street (66); Smooth independent school commuting routes; Independent walking or cycling; unsupervised outdoor play spaces (67)	•Safe living environment within the community, including good public security, traffic safety, and nighttime security (68) Medical facilities within an accessible distance to ensure health security (69)
Belongingness need	•Meaningful interactions between children and places (70) Children's daily social interactions (71) Cultural adaptation within the community, including cultural activities and educational public services (71)	•A positive neighborhood atmosphere and strong community cohesion (72) A supportive social network within the community, such as parenting mutual aid platforms (72) Facilitating positive social connections among community members, such as parenting support groups and community activities (72)
Esteem need	•Exclusive play spaces for children (73) Having appropriate spaces for freely expressing their ideas (67) Children's participation in community decision-making (74) Opportunities for community participation and diverse experiences, such as concerts (75)	•Parental engagement and feedback mechanism in the community (76) Spaces for showcasing parental skills, such as parent forums and talent exhibition venues (59)
Self-actualization need	•Providing a learning environment (70) Activities and facilities for interest development (54)	•An open community atmosphere for self-directed learning (48, 62)

#### 2.2.2 Analysis of community environmental systems—based on environmental behavior theory

The environmental system is inherently complex, encompassing not only the physical spatial environment but also social, ecological, and cultural dimensions (18–21). Addressing how to comprehensively capture the full range of environmental systems and diverse scenarios involved in the daily lives of children and parents is a key issue that needs to be addressed.

Previous research has primarily focused on single or dual-dimensional environmental characteristics in assessing residents' life satisfaction and wellbeing (22, 23). For instance, Zhou (24) evaluated child-friendly communities by examining physical space attributes such as traffic safety, environmental facilities, walkability, outdoor activity space size, and landscape diversity. Cao (22) analyzed neighborhood environmental features, assessing their impact on life satisfaction through factors such as spatial density, functional diversity, spatial design, and environmental attributes. Similarly, Salmistu and Kotval (25) developed an elderly-friendly community model by integrating physical and social environments tailored to residents' needs and preferences. While these studies provide valuable insights into supportive environmental design for specific populations, there is a lack of systematic research on comprehensive and multidimensional environmental systems. Therefore, establishing an inclusive and multidimensional environmental classification system that aligns with the needs of childcare and parenting groups is crucial for constructing an effective and adaptable CCPSS.

Environmental Behavior Theory emphasizes the interactive relationship between individual, behaviors, and the environment, asserting that behavior serves as an intermediary linking individual needs with environmental characteristics (26–28). Fundamental human needs are influenced by behavioral subsystems, and the occurrence of behaviors depends on specific environmental conditions, making the environment an inseparable component of behavioral patterns (28). For instance, social behaviors occur within social environments, which encompass not only physical spaces but also broader social interactions and support networks (19). Based on this perspective, this study extends the definition of the environment beyond a singular spatial dimension, incorporating a wide range of behavioral contexts and need characteristics.

Li (29) classified behavioral systems into five categories: personal physiological behavior, social behavior, individualistic behavior, cultural behavior, and physical environmental behavior. This classification comprehensively captures individuals' behavioral expressions within their environment, providing a theoretical foundation for refining environmental system classifications. Based on Environmental Behavior Theory, this study proposes a behavior-driven environmental system classification method, systematically aligning environmental systems with behavioral subsystems, which divides it into personal physiological environments, physical spatial environments, social spatial environments, cultural spatial environments, personalized behavioral environments.

**Figure 1:**
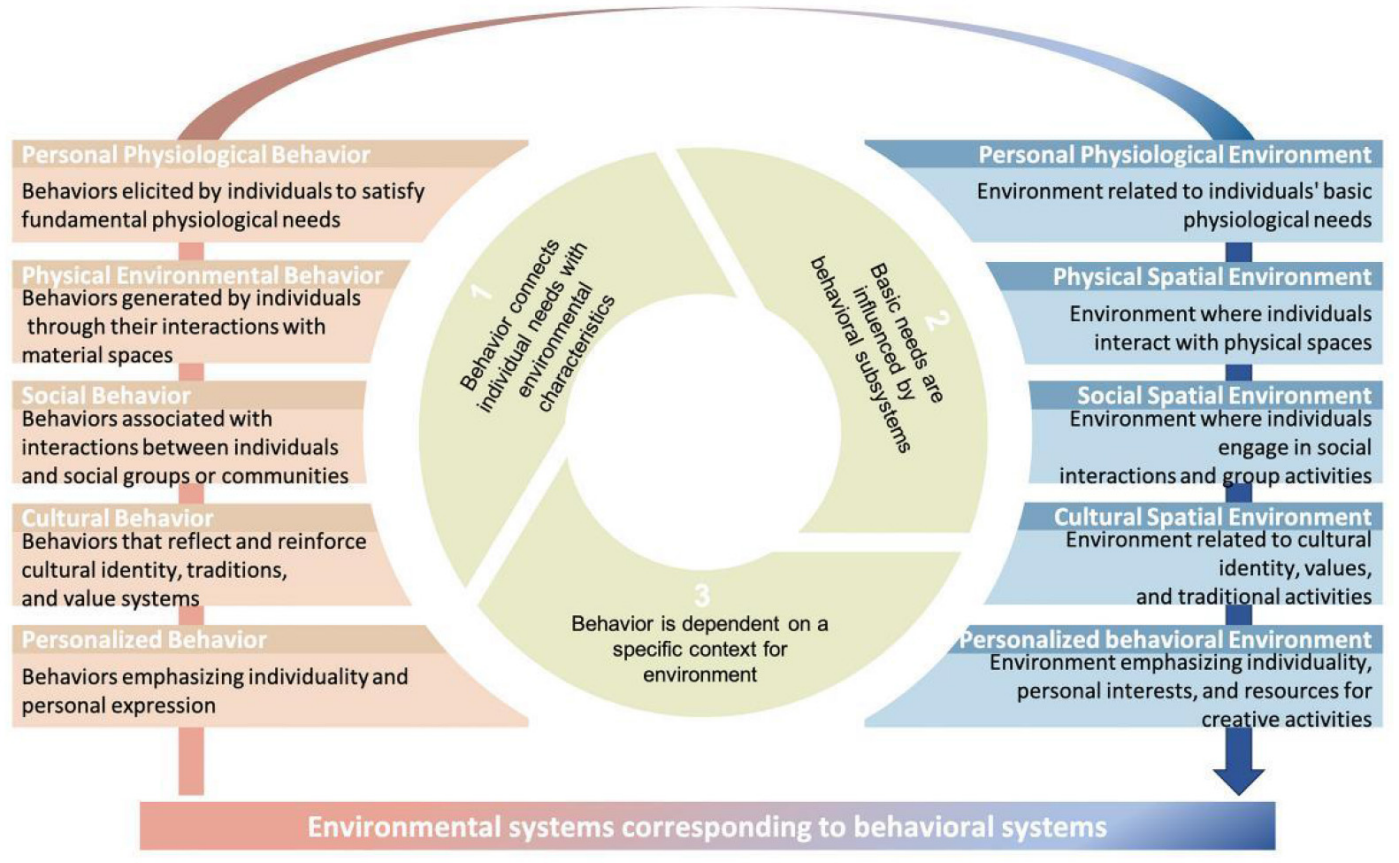
Environmental system transformation.

This classification method establishes a direct link between behavioral needs and environmental attributes, making it easier to determine how different environmental factors either support or hinder the fulfillment of children's and parents' needs. Furthermore, it expands the definition of the environment to include both tangible (e.g., physical spaces and infrastructure) and intangible (e.g., social relationships and cultural contexts) components—dimensions not fully explored in previous studies. By expanding the scope of environmental research, this classification provides a structured and scientific framework for systematically analyzing and constructing a CCPSS (Figure 1).

### 2.3 CCPSS theoretical construction

This study is grounded in P-E Fit Theory and supplemented by Environmental Behavior Theory to examine the fit between childcare and parenting needs and various environmental systems, establishes a framework for a CCPSS. The framework emphasizes the interaction between individual needs and community support environments. Environmental systems influence the satisfaction and transformation of childcare needs through the provision of basic living conditions, socio-cultural embedding, and the generation of development pathways. At the same time, the needs of children and parents continuously feed back into and reshape community environmental systems through daily activity and social participation, forming a dynamic process of need-environment interaction.

**Figure 2:**
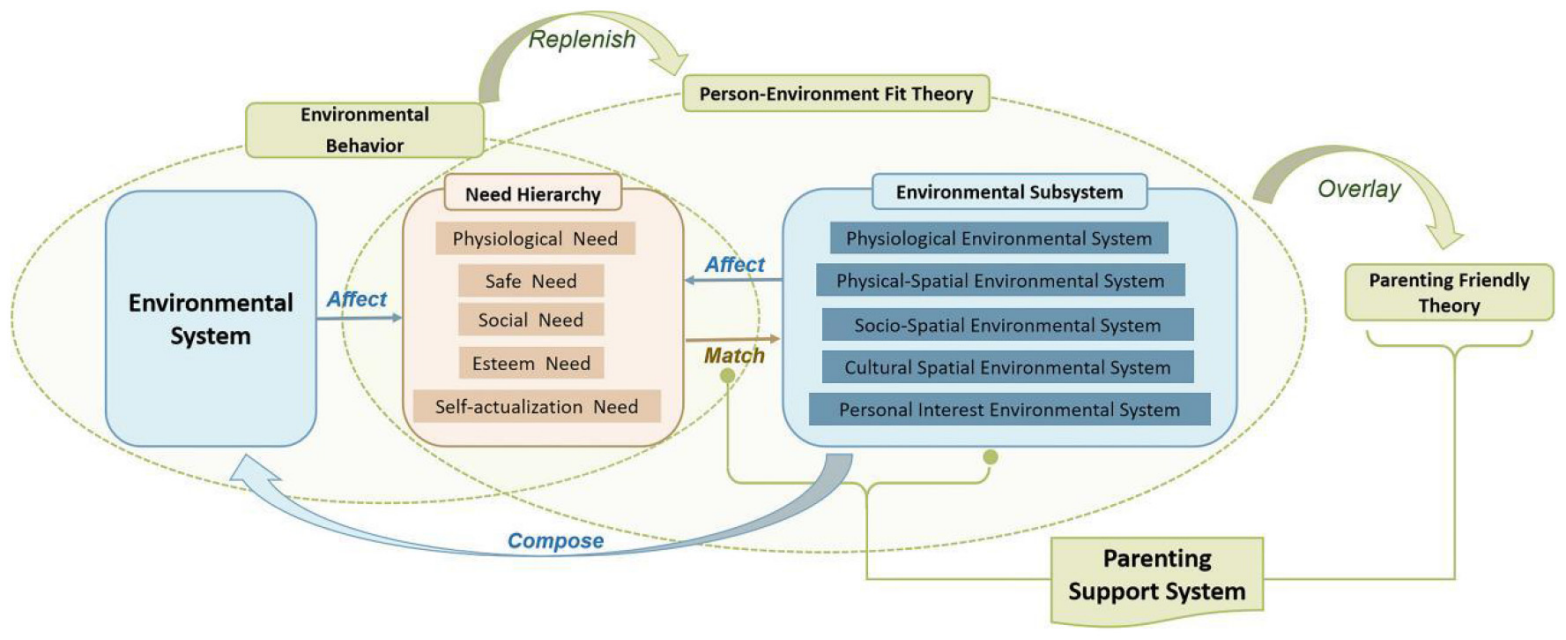
Theoretical construction of CCPSS.

Guided by this framework, the following sections analyze the correlation analysis between individual needs and community environmental systems, and further identify differences in the roles of environmental systems in meeting multi-level childcare needs through relative importance analysis. By quantifying the fit between the needs of children and parents and the community environment, this study provides a scientific foundation for optimizing the design of the CCPSS and implementing targeted interventions. As shown in Figure 2.

## 3 Materials and method

**Figure 3:**
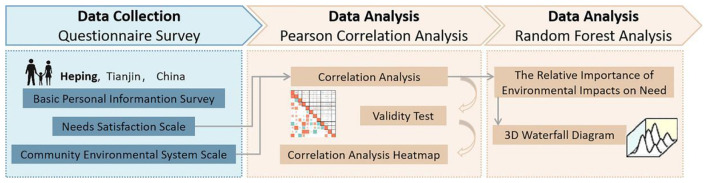
Research framework.

This study aims to examine the fit between the needs of children and parents and the community environment to develop a precise and efficient childcare and parenting support system and improve the living environment of parent-child groups. The research is structured into three phases. First, Heping District in Tianjin was selected as the study area. A questionnaire survey was designed based on hierarchical needs theory and community environmental systems to collect data separately from children and parents. The collected data were then organized and subjected to preliminary analysis. Second, Pearson correlation analysis was employed to verify the relationship between the satisfaction of childcare and parenting needs and the community environment system. Finally, random forest analysis was conducted to assess the relative importance of environmental factors in predicting the satisfaction of childcare and parenting needs. The findings provide a scientific foundation for constructing an CCPSS (Figure 3).

### 3.1 Study area and object

**Figure 4:**
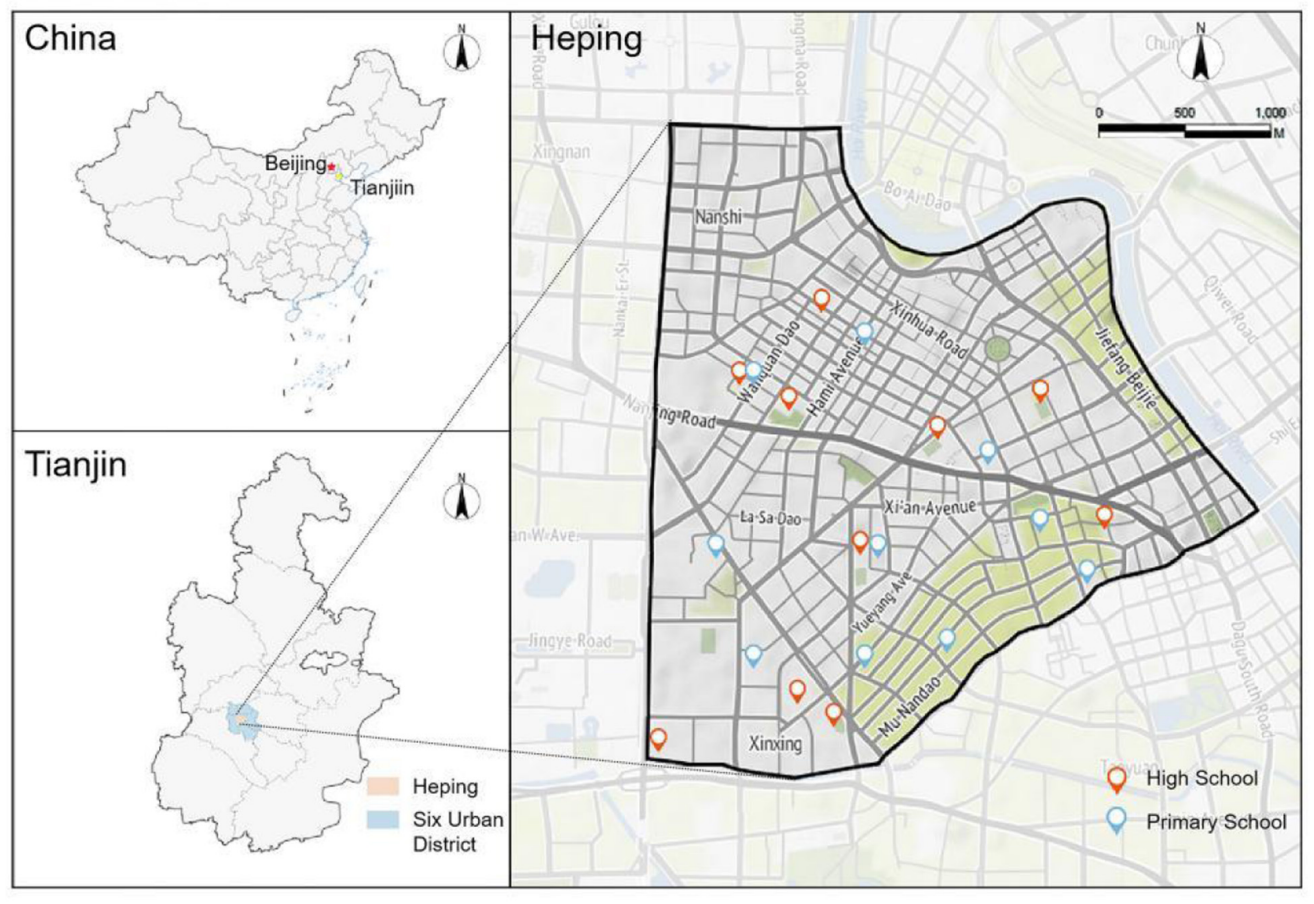
Research area.

This study selected Heping District in Tianjin as the research area. As a metropolitan city in northern China, Tianjin serves as a typical and representative case for research on community renewal. Heping District, one of the central urban districts of Tianjin, has a long history of urban construction, with residential communities built across different periods, resulting in a complex demographic structure and significant challenges in community renewal. Moreover, Heping District is home to several key municipal middle and high schools, along with diverse educational and recreational resources that support the holistic and high-quality development of school-age children. The district's strong educational infrastructure creates an environment conducive to children's comprehensive growth (Figure 4).

Due to these factors, and under the influence of China's “school district housing” policy, the area has attracted a high concentration of families with school-age children. According to data from China's Seventh National Population Census (2020), Heping District has the highest proportion of children aged 5–14 among Tianjin's six core districts, accounting for 14.56% of the total population. The high density of children and parents further exacerbates the conflict between childcare needs and the limited resources available in old communities. Therefore, selecting Heping District as the study area is both representative and methodologically relevant for investigating childcare and parenting support systems in urban settings.

### 3.2 Data collection: questionnaire survey on children's and parents' needs and community environment systems

This study employs a questionnaire survey to collect data for analyzing the relationship between needs satisfaction among children and parents and the community environment systems. Separate questionnaires were designed for children and parents using a five-point Likert scale (1 = very dissatisfied, 5 = very satisfied), a format widely used for its ease of comprehension and ability to enhance response reliability and validity (30, 31).

The questionnaire consists of three sections: (1) demographic information, (2) a needs satisfaction scale, and (3) a community childcare and parenting support environment system evaluation scale. The demographic section collects essential participant data, including age, gender, family composition, and residential community information and so on. The needs satisfaction scale, developed based on Maslow's hierarchy of needs theory, assesses the extent to which respondents' physiological, safety, belonging, esteem, and self-actualization needs are met within the community. The CCPSS evaluation scale, developed based on Environmental Behavior Theory, examines five key environmental dimensions: personal physiological environments, physical spatial environments, social spatial environments, cultural spatial environments, and personalized behavioral environments. Item statements for each scale were developed through a systematic review of literature related to child development, community support, and parenting needs, and further designed in reference to specific environmental characteristics associated with childcare in community settings, to maintain conceptual alignment between theoretical constructs and measurement dimensions and reflect the multidimensional structure of needs and environmental support systems.

Considering differences in age and comprehension between children and parents, separate questionnaires were designed for each group. While both versions were based on the same theoretical dimensions, age-appropriate adjustments were made in wording and presentation format. For the child questionnaire, pinyin annotations were added, and emotive symbols were used to indicate levels of satisfaction. These modifications were introduced to improve comprehension and response accuracy among child participants.

The survey was conducted between December 2023 and January 2024. Questionnaires were distributed in communities surrounding primary and secondary schools in Heping District, Tianjin. The questionnaire survey was divided into offline and online methods. The online questionnaire was developed using Wenjuanxing (Chinese online questionnaire survey website) and was disseminated exclusively through residential WeChat groups within communities in Heping District to ensure that respondents were located within the defined study area. A total of 207 valid questionnaires were collected, including 96 from children and 101 from parents or parents.

Given that the scales were specifically developed for the community childcare and parenting support context, exploratory factor analysis (EFA) was conducted separately for the children and parents samples to examine the dimensional structure and internal consistency of the instruments. To explore the dimensional structure of the newly developed scales, Principal Component Analysis (PCA) with Varimax rotation was conducted. The number of factors retained was determined with reference to theoretical expectations and the eigenvalue-greater-than-one criterion. Items were retained if their factor loadings were no less than 0.50 and no substantial cross-loadings were observed. The results generally supported the theoretically hypothesized multidimensional structure, providing preliminary support for the operationalization of the constructs. Although both questionnaires were developed under the same theoretical framework, exploratory factor analyses were conducted separately for children and parents to account for differences in developmental stage and cognitive capacity.

This study received ethical approval from the Ethics Committee of Tianjin University. Prior to participation, detailed information and consent forms were provided to parents or legal guardians of all child participants. Written informed consent was obtained from all participants, ensuring their voluntary participation in accordance with ethical guidelines.

### 3.3 Data analysis: Pearson correlation analysis of community children's and parents' needs and environmental systems

This study employed Pearson correlation analysis to examine the relationship between different levels of children and parents needs and the community environment system. The primary objective is to identify key factors influencing childcare and parenting-friendliness and to provide a foundation for subsequent analyses of the significance of community environmental systems in meeting these needs.

Pearson correlation analysis is a statistical method used to assess the degree of linear association between two random variables, measuring both the strength and direction of their relationship. In this study, Pearson correlation analysis was applied to quantitatively evaluate the linear correlations between the satisfaction of childcare and parenting needs and the community environment system. The calculation formula is as follows:


$$\begin{array}{l} r=\frac{\sum \left(x_{i}\bar{x}\right)\left(y_{i}\bar{y}\right)}{\sqrt{{\sum \left(x_{i}\bar{x}\right)}^{2}{\sum \left(y_{i}\bar{y}\right)}^{2}}} \end{array}$$

where:

r represents the correlation coefficient between the level of individual needs and the environmental subsystem.

X_i_ denotes the satisfaction level of individual needs, while $\bar{x}$ represents the mean value of this variable.

Y_i_ indicates the satisfaction level of environmental subsystem, and $\bar{y}$ represents the mean value of this variable.

### 3.4 Data analysis: assessing the impact of environmental systems on children and parents needs satisfaction using random forest relative importance analysis

Random forest is a machine learning method widely applied in urban studies and is commonly used to investigate complex issues such as life satisfaction, transportation analysis, and urban correlations (32, 33). Compared with other machine learning techniques such as support vector machines (SVM), artificial neural networks (ANN), and gradient boosting decision trees (GBDT), random forest offers greater interpretability in identifying variable importance, making it suitable for this research for the following three reasons.

First, random forest quantifies the relative contribution of each predictor by calculating variable importance measures, thereby providing a basis for prioritizing environmental subsystems. In contrast, although ANN and SVM demonstrate strong predictive performance, they offer limited interpretability regarding the influence of individual variables (34). Second, random forest is robust against multicollinearity and effectively handles high-dimensional data, which is particularly advantageous when ranking the importance of multiple highly correlated environmental factors influencing individual wellbeing (33). Finally, unlike regression models, random forest does not rely on strict distributional assumptions, allowing greater flexibility in analyzing diverse environmental and behavioral variables.

Random forest regression was implemented in Python using the scikit-learn library. The five community environmental subsystem dimensions were specified as independent variables, and the satisfaction levels of different need tiers among children and parents were treated as dependent variables. The model was configured with 500 decision trees, and a fixed random seed was set to ensure reproducibility; all other parameters were retained at their default settings. To assess the stability of the importance ranking, additional tests were conducted by varying the number of trees (100–500). It should be noted that the variable importance values derived from the random forest reflect the relative contribution of each environmental dimension in predicting need satisfaction. The results should therefore be interpreted as indicating associative relationships and priority rankings, rather than causal effects of environmental systems on need satisfaction.

This research employs random forest-based relative importance analysis to examine the relationship between parenting group needs and community environmental systems. The analysis identified the relative weights of different environmental subsystems in predicting need satisfaction among children and parents, thereby providing a basis for prioritizing interventions in the optimization of the CCPSS and the formulation of targeted childcare and parental support policies.

The calculation formula is as follows:


$$\begin{array}{l} I\left(X_{j}\right)=\frac{1}{T}\overset{T}{\underset{t=1}{\sum }}\underset{n\in N_{t},v=n_{j}}{\sum }\Delta i_{n},t \end{array}$$

Where:

I(X_j_) represent the relative importance score of variable X_j_,

T is the total number of trees in the random forest,

N_t_ is the set of all nodes in tree t,

v =n_j_ indicates that node n splits on variable X_j_,

ΔI_n_, t is the decrease in impurity (e.g., Gini impurity or entropy) at node n in tree t.

## 4 Results

### 4.1 Reliability and validity assessment of questionnaire

To ensure the reliability and validity of the survey, statistical analyses were conducted on the Personal Needs Satisfaction Scale and the Community Childcare and Parenting Support Environment System Assessment Scale. The Cronbach's alpha coefficients for the parent version of the Personal Needs Satisfaction Scale and the Community Childcare and Parenting Support Environment System Assessment Scale were 0.825 and 0.866, respectively. For the child version, the Cronbach's alpha coefficients were 0.909 and 0.962, respectively. These values demonstrate a high level of stability and consistency in the scales used in this research.

Additionally, the validity of the scales for both parents and children was assessed. The Kaiser–Meyer–Olkin (KMO) values for the parent version of the Personal Needs Satisfaction Scale and the Community Childcare and Parenting Support Environment System Assessment Scale were 0.824 and 0.859, respectively. For the child version of the scales, the KMO values were 0.765 and 0.847, respectively. Bartlett's test of sphericity was significant, confirming that the designed questionnaires adequately measure the intended aims, validating the research instruments.

On this basis, EFA was conducted separately for the children and parent samples. Item retention criteria were set at factor loadings ≥0.50 with no substantial cross-loadings. The results indicated that the need satisfaction scale yielded a five-factor structure in both the children's and parents' samples, corresponding to the dimensions of physiological, safety, belongingness, esteem, and personal development needs. The cumulative variance explained was 84.757% for the children's sample and 67.268% for the parent sample. Factor loadings ranged from 0.525 to 0.905 for children and from 0.511 to 0.649 for parents, all exceeding the predefined threshold. Similarly, the community environmental system scale extracted five factors in both samples, corresponding to the dimensions of personal physiological, physical spatial, social spatial, cultural spatial, and personalized behavioral environments. The cumulative variance explained reached 92.017% for children and 71.111% for parents, with factor loadings ranging from 0.545 to 0.953 in the children's sample and from 0.547 to 0.899 in the parent sample.

**Figure 5:**
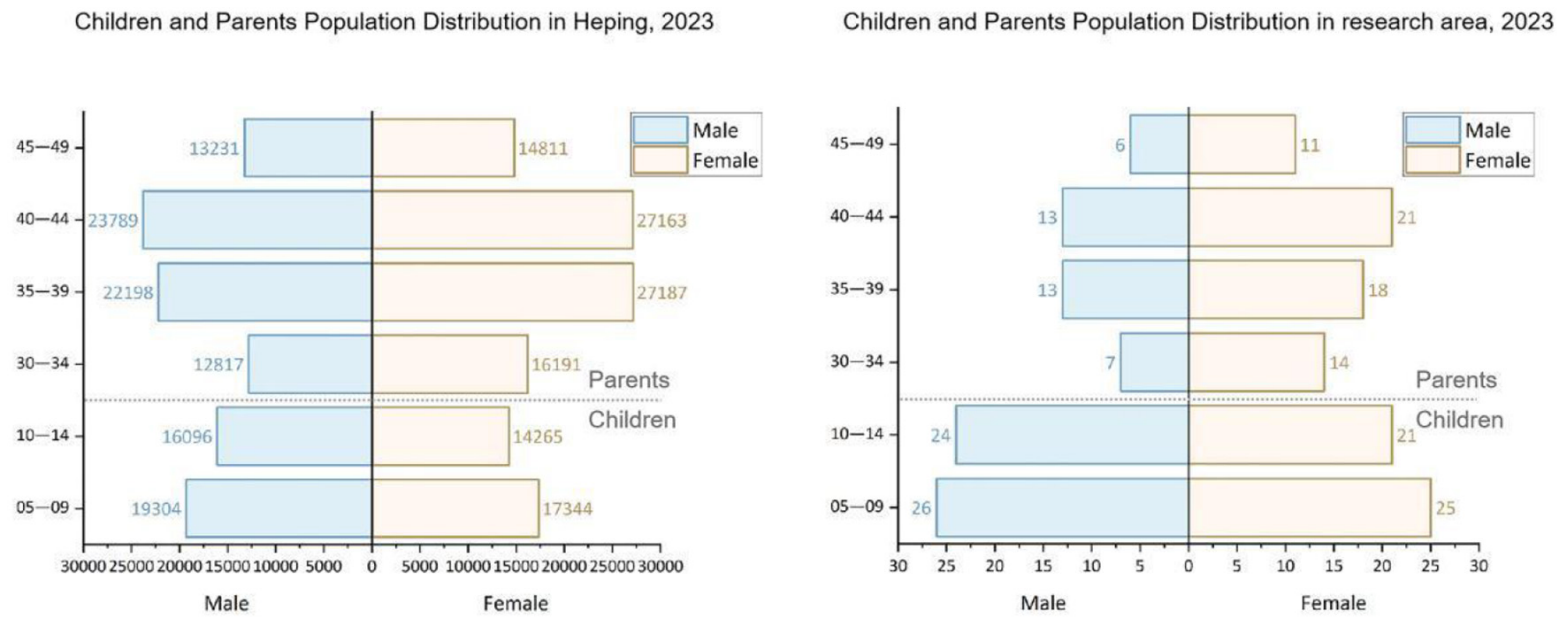
Comparison of age distribution of Tianjin Heping and the research area.

Moreover, an initial analysis of the surveyed children and parents was conducted. The gender distribution of child respondents was 60% male and 40% female, with an age range of 5–14 years. Among parent respondents, 40% were male and 60% were female, with the majority aged 30–49 years. By comparing these demographic distributions with the 2023 Tianjin Heping District Statistical Yearbook, the study determined that the gender and age distribution of respondents closely aligns with the overall population composition of Tianjin. This similarity indicates that the sample is representative and provides a reliable basis for further analysis (Figure 5).

### 4.2 Correlation between child and parent needs satisfaction and the community environment system

This study employed Pearson correlation analysis to examine the relationship between different levels of child and parent needs satisfaction and the community environment system. The results indicate a significant positive correlation across all variables, with *p*-values below 0.05, confirming the robustness of the associations. To enhance interpretability, the correlation analysis results were visualized using a heatmap, providing an intuitive representation of the relationships between needs satisfaction levels and the community environment system.

#### 4.2.1 Correlation analysis between child needs satisfaction and the community environment system

**Figure 6:**
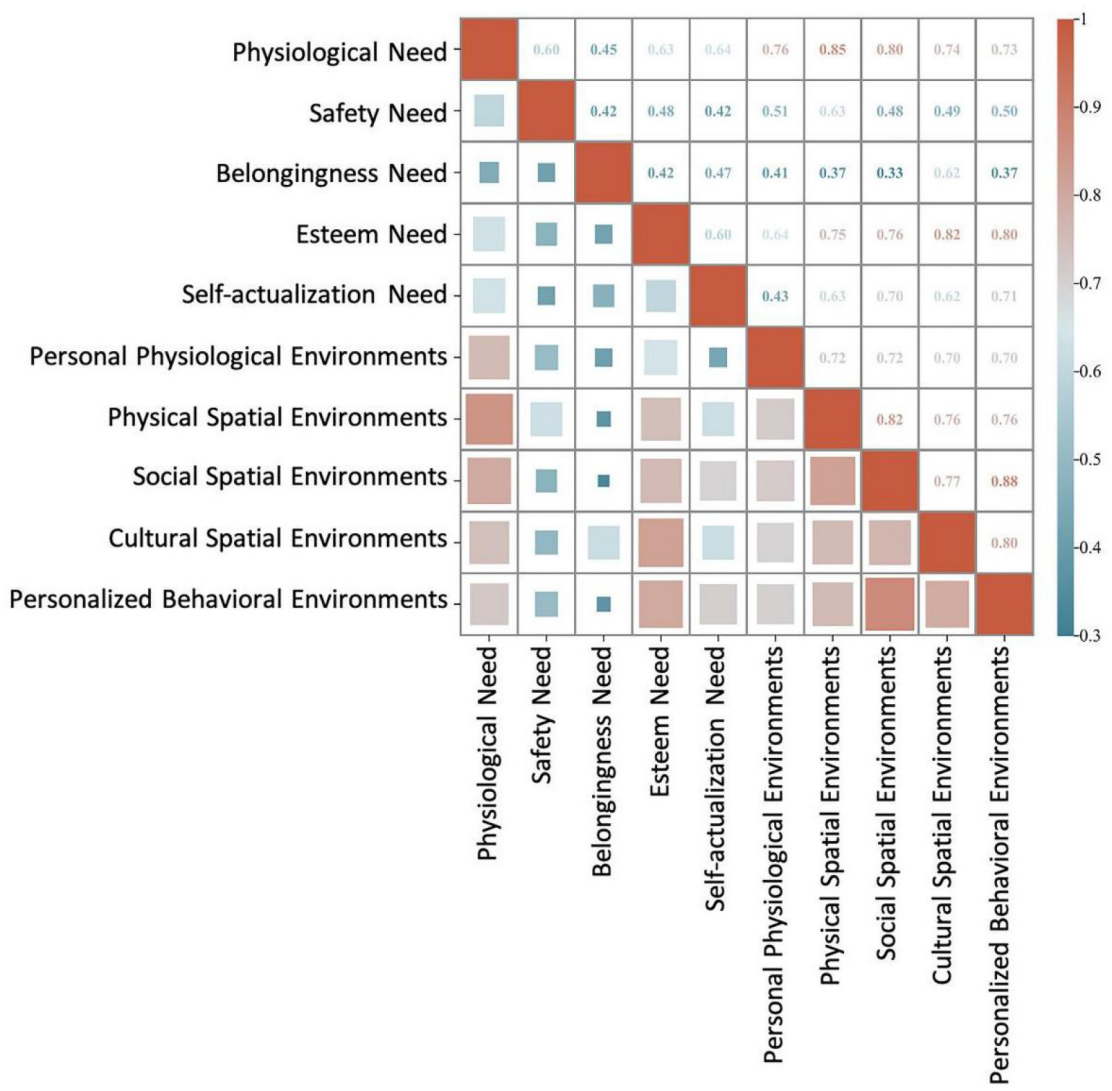
Pearson correlation analysis between children needs satisfaction and the community environment system.

The analysis results show a significant positive correlation between child satisfaction across different need levels and community environment system (Figure 6). Overall, physiological needs and esteem needs demonstrate the strongest correlation with the community environment system compared to other need levels. Specifically:

- Physiological needs show the highest correlation with the physical spatial environment (*r* = 0.854, *p* < 0.01), suggesting that physical infrastructure and space are key factors in meeting children's physiological needs. Additionally, a strong correlation is observed with the personal physiological environment (*r* = 0.759).
- Safety needs are significantly correlated with the physical spatial environment (*r* = 0.630) and the personal physiological environment (*r* = 0.507), indicating that these environmental factors play a supportive role in ensuring children's safety.
- Belongingness and esteem needs are most strongly correlated with the cultural spatial environment (*r* = 0.620 and *r* = 0.821, respectively), highlighting the crucial role of cultural atmosphere in fulfilling children's sense of belonging and respect. Additionally, esteem needs also show a high correlation with the social spatial environment (*r* = 0.760).
- Self-actualization need exhibit the strongest correlation with the social spatial environment (*r* = 0.697), followed by the personalized behavioral environment (*r* = 0.711), indicating that a supportive social atmosphere and personalized interest-based activities are essential for children's growth.

#### 4.2.2 Correlation analysis between parents needs satisfaction and the community environment system

**Figure 7:**
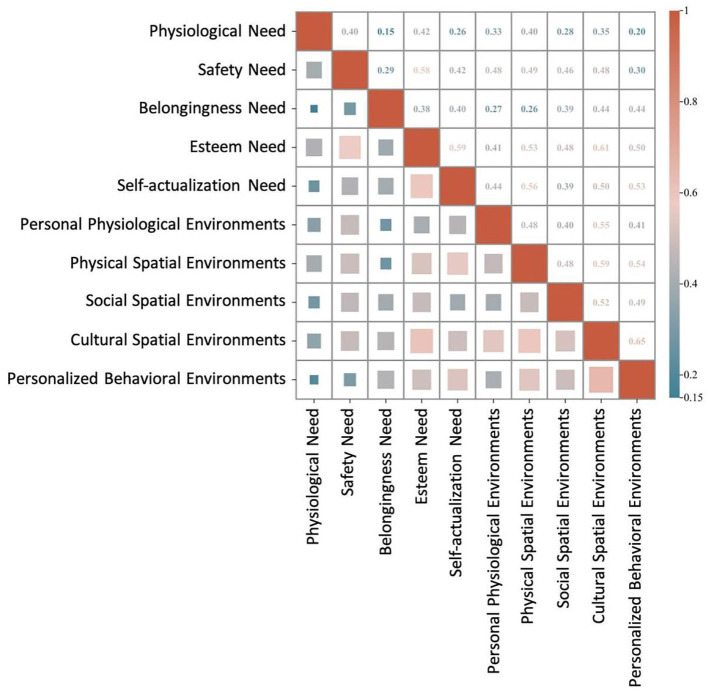
Pearson correlation analysis between parents needs satisfaction and the community environment system.

The correlation between parents' needs satisfaction and the community environment system exhibits notable differences compared to children's satisfaction. Specifically, esteem needs and self-actualization needs demonstrate strong correlations with the community environment, whereas physiological needs exhibit the weakest correlation (Figure 7). The detailed analysis is as follows:

- Physiological and safety needs show strong correlations with both the physical spatial environment (*r* = 0.395 and *r* = 0.492, respectively, *p* < 0.01) and the personal physiological environment (*r* = 0.326 and *r* = 0.480, respectively, *p* < 0.01), indicating that these environmental dimensions play a crucial role in fulfilling parents' physiological and safety needs.
- Belongingness needs exhibit a significant positive correlation with the cultural spatial environment (*r* = 0.439), suggesting that the cultural atmosphere within the community fosters a sense of belonging among parents. Additionally, belongingness needs also show a positive correlation with the social spatial environment (*r* = 0.392), further emphasizing the role of social connections in fulfilling this need.
- Esteem needs are strongly associated with both the cultural spatial environment (*r* = 0.607) and the physical spatial environment (*r* = 0.525), indicating that community cultural activities and cultural spaces significantly contribute to fulfilling parents' esteem needs.
- Self-actualization need exhibit the strongest correlation with the personalized behavioral environment (*r* = 0.531), followed by the cultural spatial environment (*r* = 0.496). This suggests that personalized spaces for interest development and cultural environments play a critical role in supporting parents' personal growth.

The analysis of inter-environmental correlations further validates the interdependent relationships among different environmental dimensions. As observed in the correlation analysis for children, the cultural spatial environment and social spatial environment exhibit a high correlation (*r* = 0.771), suggesting that these two environmental factors may function synergistically in providing supportive resources. Similarly, the personalized behavioral environment is strongly correlated with the social spatial environment (*r* = 0.878), suggesting that social support and opportunities for participation provide essential contextual conditions for the development of interest-based activities.

In summary, significant positive correlations are observed between children and parents' needs satisfaction and the community environment system, which provides preliminary support for the rationality of constructing a parenting support system based on the P-E fit theory. However, the magnitude of correlation coefficients alone is insufficient to determine the relative importance of environmental factors. Therefore, the subsequent section applies random forest importance ranking analysis to further investigate the key environmental factors influencing different levels of need satisfaction, providing a more reliable scientific basis for optimizing the support system.

### 4.3 Relative importance of environmental subsystems in meeting the needs of children and parents

To further identify the relative contributions of different environmental subsystems in explaining variations in need satisfaction among children and parents, a random forest model was applied to rank the importance of each environmental dimension. The results indicate that the relative importance rankings of environmental subsystems differ substantially between the children's and parents' models, suggesting that the roles of community environmental systems vary across groups and across different levels of need satisfaction.

#### 4.3.1 Relative importance of environmental subsystems in meeting children's needs

**Figure 8:**
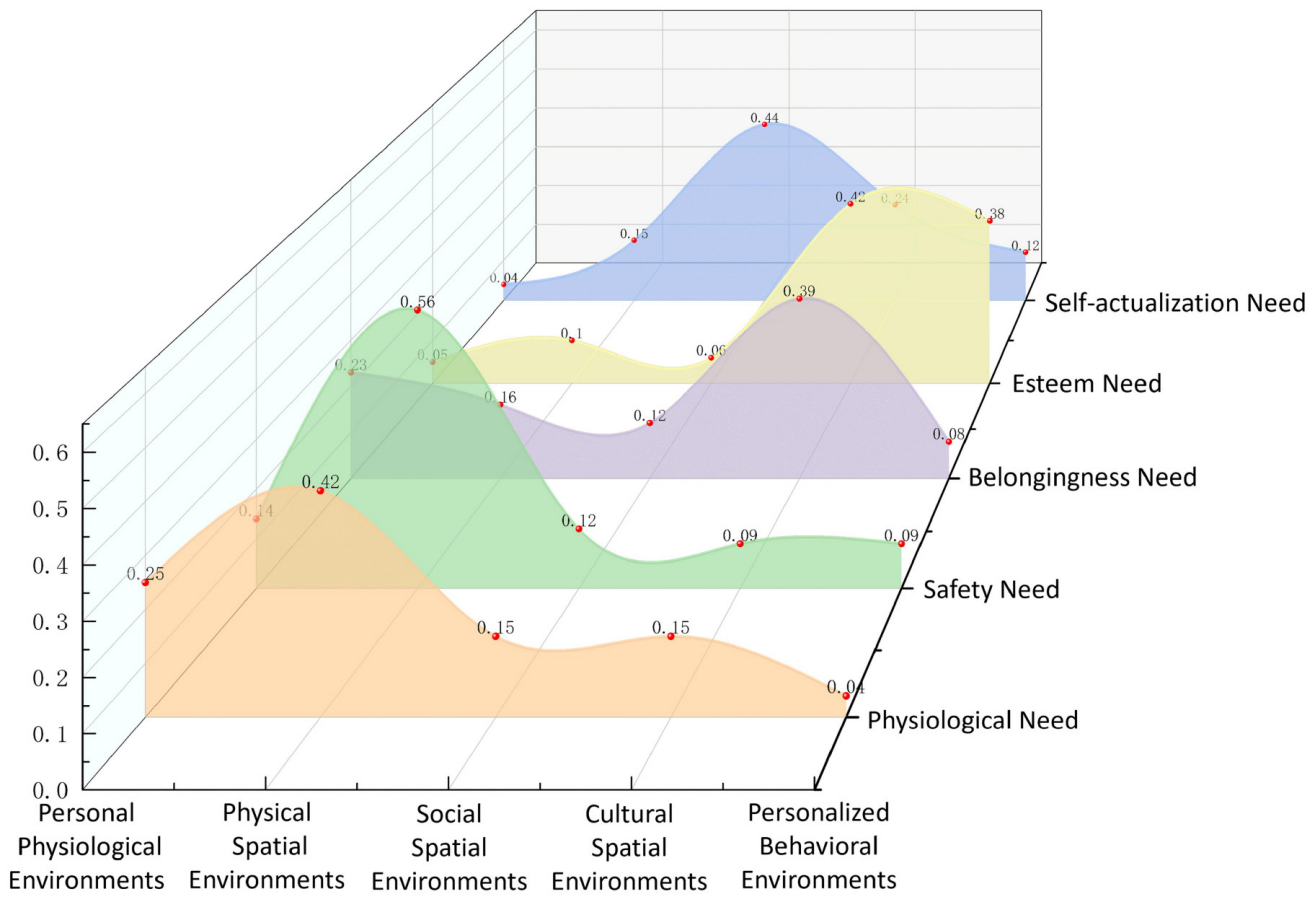
Relative importance of environmental subsystems in meeting children's needs.

**Figure 9:**
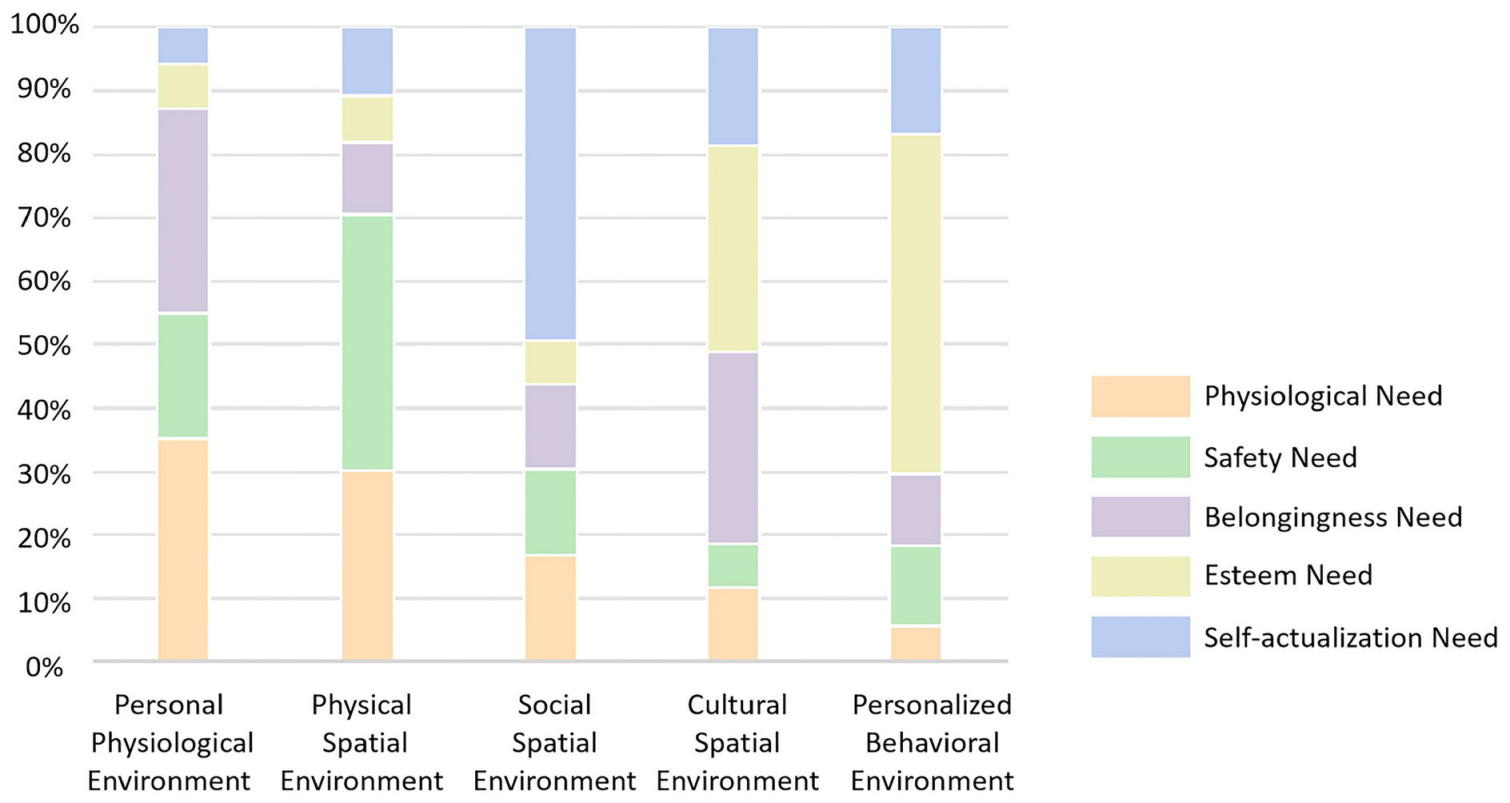
Relative importance of environmental subsystems (children).

The analysis of the relative importance of environmental subsystems reveals distinct variations in their relative contributions across different levels of children's needs (Figures 8, 9).

For physiological needs, the physical spatial environment accounts for the largest proportion of importance, representing 42% of the total importance, underscoring the critical role of physical spaces and infrastructure in the community. The personal physiological environment follows with a relative importance of 0.25, indicating that environments, such as the homes, childcare centers, and other caregiving environments, that meet children's physiological needs play an important ancillary role within the support system. In contrast, the personalized behavioral environment contributes minimally, accounting for only a negligible proportion of physiological need satisfaction.

For safety needs, the physical spatial environment again accounts for the highest share, at 56%, substantially exceeding other environmental factors. This result highlights that community infrastructure and spatial planning are fundamental in ensuring children's safety. The remaining four environmental subsystems have comparatively minor and balanced influences, with cultural spatial environment exhibiting the lowest importance at only 0.09.

For belongingness needs, the cultural spatial environment accounts for the largest share, contributing 39% to the total importance, which aligns with expectations. This finding emphasizes the significant role of community cultural atmosphere and resources in fostering a sense of belonging among children. Notably, the personal physiological environment ranks second (0.23), suggesting that a well-established family environment and free movement spaces provide essential support for children's sense of belonging. The personalized behavioral environment has the least impact, with only 0.08 importance.

For esteem needs, the cultural spatial environment again accounts for the highest proportion of importance (0.42), reaffirming its central role in developing children's self-esteem through community culture and educational facilities. Unlike other need levels, the personalized behavioral environment ranks second for the first time, with a relatively high importance of 0.38, underscoring the significance of interest-based activities in fostering children's self-respect and esteem. Other environmental factors have comparatively minor influences on esteem needs.

For self-actualization needs, the social spatial environment accounts for the largest proportion of importance, at 0.44, highlighting its pivotal role in children's personal growth and development. The cultural spatial environment follows, accounting for 0.24, demonstrating the positive influence of cultural resources and facilities on children's personal development. Meanwhile, the personalized behavioral environment has the least impact, with an importance of only 0.04.

#### 4.3.2 Relative importance of environmental subsystems in meeting parents' needs

**Figure 10:**
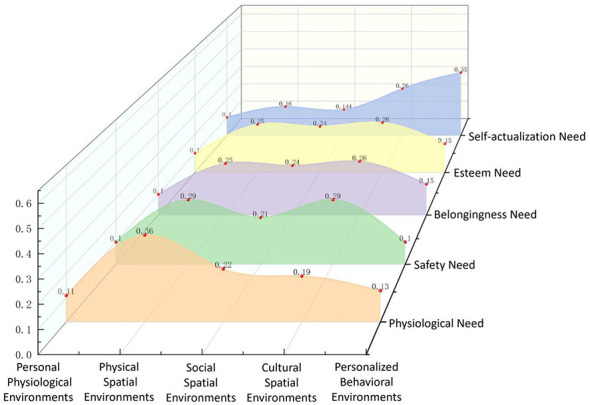
Relative importance of environmental subsystems in meeting parents' needs.

**Figure 11:**
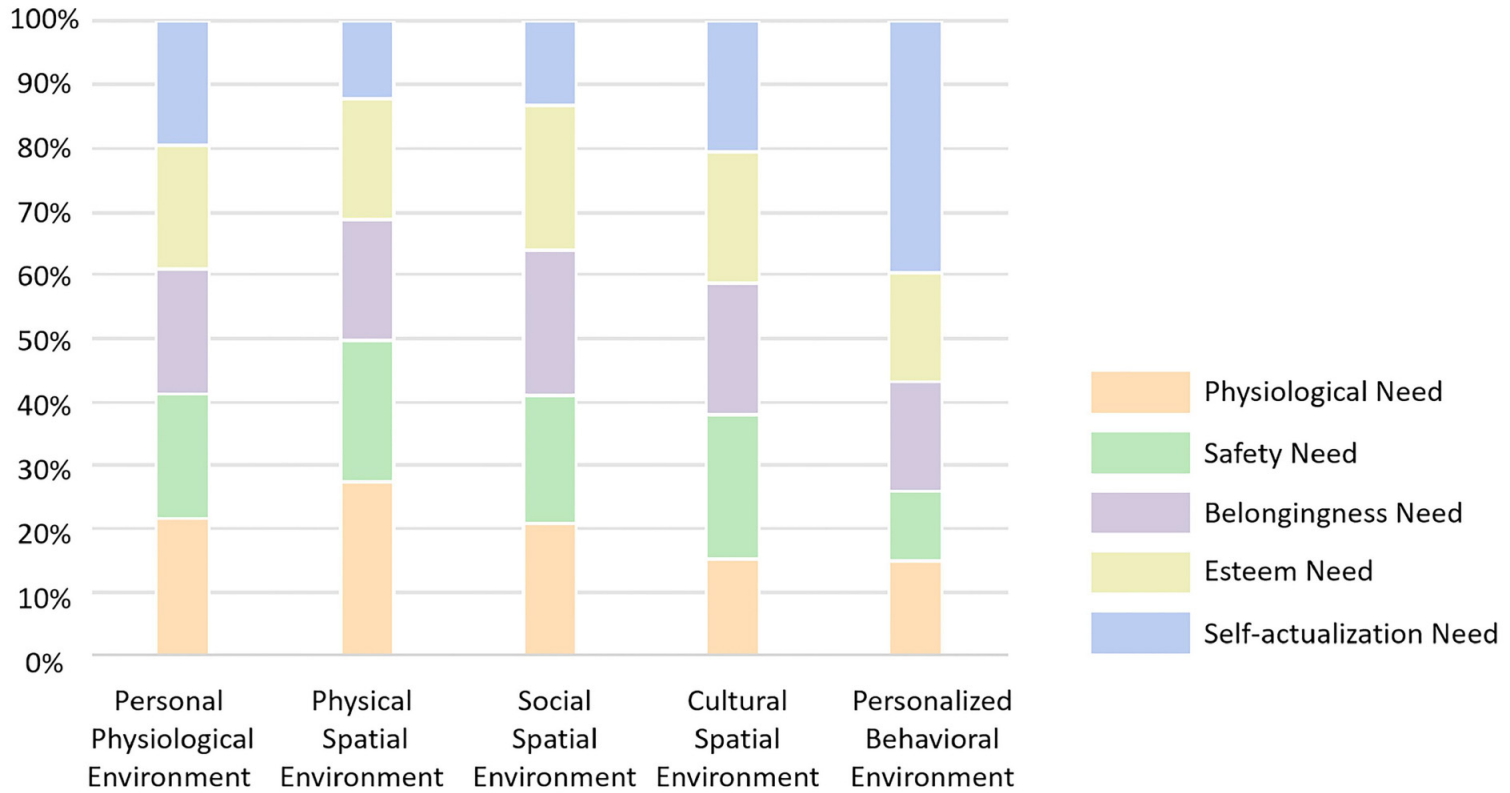
Relative importance of environmental subsystems (parents).

The analysis of environmental systems' relative importance in fulfilling parents' needs reveals distinct patterns compared to children (Figures 10, 11).

For physiological needs, the physical spatial environment remains the most critical factor, contributing 36% to the overall importance. Notably, the social spatial environment ranks second (0.22), highlighting the significant role of social support networks and neighborhood interactions in meeting physiological needs. In contrast, the personalized behavioral environment has a limited impact, accounting for only 0.11.

For safety needs, while similar to children's results, the physical spatial environment (0.29) remains the most important factor. However, cultural spatial environment (0.29) is equally influential, indicating that both physical space and cultural atmosphere play a crucial role in shaping parents' perception of safety. Meanwhile, the personal physiological environment has the weakest influence, contributing only 0.10.

For belongingness needs, the cultural spatial environment emerges as the most significant factor, accounting for 26%, followed by the physical spatial environment (0.25%), suggesting that a well-developed cultural atmosphere and accessible public spaces play an essential role in enhancing parents' sense of belonging. In contrast, the personal physiological environment has the least impact, contributing only 0.10.

For esteem needs, the cultural spatial environment (0.26%) ranks highest, albeit by a narrow margin. The physical spatial environment (0.25%) and social spatial environment (0.24%) follow closely, indicating that both physical spaces and social interactions significantly contribute to the fulfillment of esteem needs. The personal physiological environment has the weakest impact, with only 0.10 importance.

For self-actualization needs, the personalized behavioral environment holds the highest relative importance (0.35%), underscoring the crucial role of interest-based activity spaces in personal growth and skill development for parents. The cultural spatial environment (0.26%) ranks second, suggesting that community cultural settings play an essential role in supporting parents' personal development. The personal physiological environment has the least influence, contributing only 0.10.

To assess the stability of the priority ranking results, the study validated them by varying the number of decision trees (100–500). The relative importance rankings remained largely consistent across model specifications, indicating that the priority ordering derived from the model was robust.

## 5 Discussion

### 5.1 Exploring the impact mechanism of key environmental determinants on need satisfaction in CCPSS

The findings suggest that the environmental factors most crucial to meeting the needs of both children and parents exhibit consistency at the basic need level but diverge significantly at higher-level needs. These high-importance factors operate across need hierarchies by providing foundational material conditions, embedding individuals within socio-cultural contexts, and creating opportunities for development, thereby exerting differentiated effects on distinct need levels among children and parents.

#### 5.1.1 The role of physical spatial environments in meeting the basic needs of children and parents

The physical spatial environment of a community plays a fundamental role in fulfilling the physiological and safety needs of both children and parents. Physical spatial environments serve as the material foundation for fulfilling the basic needs of children and parents by providing accessible activity spaces, safety facilities, and essential living support conditions.

For children, prior research indicates that elements of the built environment, including building design, floor area ratio, green space ratio, entertainment facilities, community parks, and home environments, significantly impact early childhood development, physical health, and psychological wellbeing (35, 36). For instance, well-designed architectural spaces and accessible green areas have been found to enhance children's physical growth, physical activity engagement, and mental health. Additionally, community outdoor play facilities, particularly varied play areas, natural elements, and safety-enhancing building materials, contribute to improving children's perceived safety (37). Moreover, traffic environments within a community directly affect children's mobility safety and autonomy in outdoor activities (38).

For parents, safe, pedestrian-friendly, and functionally diverse physical environments—including green spaces, multifunctional areas, barrier-free streets, and well-developed infrastructure—have been shown to significantly enhance comfort and perceived safety (39–42). Such environmental attributes not only reduce health risks such as obesity and cardiovascular diseases but also encourage outdoor recreational activities, indirectly supporting parenting behaviors and overall wellbeing.

These findings underscore the fundamental role of physical spatial environments in shaping childcare and parenting experiences, making them a critical determinant in fulfilling the basic needs of families within the CCPSS framework.

#### 5.1.2 The role of community cultural environment in fulfilling belonging and esteem needs of children and parents

The community cultural spatial environment plays a crucial role in fulfilling the belonging and esteem needs of children and parents. Shared cultural atmospheres and educational resources create common social contexts in which individuals gain recognition and a sense of belonging through interaction.

For children, a shared cultural background within the community can alleviate social anxiety and strengthen belongingness (43). Additionally, a well-developed cultural spatial environment and higher levels of educational provision contribute to enhanced self-efficacy, thereby facilitating the fulfillment of esteem needs (44). Studies have shown that within communities where household economic conditions are generally similar, children's access to educational and cultural resources is positively correlated with the development of self-esteem and subsequent social mobility (44).

For parents, a cohesive cultural spatial environment and shared cultural atmosphere foster trust, safety, support, and long-term interpersonal relationships among groups of adults (45). The provision of culturally enriched community spaces and participation-oriented cultural programs for parents can enhance engagement, reinforce belonging, and contribute to the fulfillment of esteem needs.

#### 5.1.3 Environmental factors influencing the self-actualization needs of children and parents

The key environmental factors influencing the fulfillment of self-actualization needs differ between children and parents. However, all support individuals' long-term development by creating opportunities for participation and capacity enhancement.

For children, the community social spatial environment emerges as a key determinant of self-actualization need. A supportive social environment is one that conveys a sense of care, wellbeing, security, neighborhood cohesion, and belonging, which enables children to better utilize community resources. This, in turn, encourages active participation in community activities, supports socialization processes, and fosters personal development across developmental stages (46).

For parents, the personalized behavioral environment within the community is a significant factor in achieving self-actualization. While self-efficacy and a well-established sense of self-worth are essential drivers of adult development, the availability of a supportive environment for pursuing personal interests remains critical for knowledge expansion and value realization (47). Additionally, Social Cognitive Theory underscores the importance of external support environments in shaping individual behavior and personal growth (48).

In summary, the physical spatial environment is fundamental in meeting the physiological and safety needs, while the cultural spatial environment is essential for fulfilling social and esteem needs. In contrast, the social spatial environment and personalized behavioral environment serve as the core support systems for fulfilling the self-actualization needs of children and parents, respectively.

### 5.2 Exploring the impact mechanism of secondary environmental determinants on need satisfaction in CCPSS

Secondary environmental factors play a crucial supporting role in fulfilling the needs of children and parents, influencing individuals' experiences and interactions with the environment, and should be given significant consideration in constructing an effective CCPSS. Unlike the key environmental determinants identified earlier, the influence of secondary environmental factors on need satisfaction in the community exhibits unexpected results.

#### 5.2.1 Analysis of secondary environmental system influencing children's need satisfaction

The personal physiological environment, personalized behavioral environment, and cultural spatial environment function as secondary determinants in shaping children's need satisfaction, each playing an essential supportive role at different need hierarchies.

The personal physiological environment significantly impacts children's physiological, safety, and belonging needs. Due to their physiological characteristics, children exhibit heightened sensitivity to micro-scale physical elements, such as colors and sounds. These multisensory stimuli substantially influence children's experiences, social interactions, and perceptions of safety in their surroundings (36). Consequently, the design of play spaces and mobility environments that align with children's physiological traits, behavioral patterns, and experiential needs can effectively reduce stress, enhance safety, and facilitate social adaptation (49–51).

The personalized behavioral environment and cultural spatial environment are particularly crucial for fulfilling children's esteem and self-actualization needs. Self-appreciation is fundamental to an individual's ability to integrate into society and adapt to life. Environments that support interest cultivation contribute directly to self-esteem formation and the fulfillment of esteem-related needs. Participation in activities such as music and art can enhance children's self-identity, laying the foundation for better social adaptation (52, 53).

Additionally, while children's personal development preferences are primarily driven by intrinsic motivation, the community, as the most open and accessible environment for children, plays a critical role. The cultural spatial environment, with its diverse cultural resources, educational activities, and cultural atmosphere, offers abundant opportunities and likelihood for children's autonomous development and strongly influences the realization of their self-actualization needs (54).

#### 5.2.2 Analysis of secondary environmental system influencing parents' need satisfaction

For the fulfillment of parents' basic needs (physiological and safety needs), an unexpected finding was the significant supporting role of the social spatial environment and cultural spatial environment. These two environmental dimensions influence individual behavioral patterns, subsequently shaping perceptions of daily life and safety (55). Recognizing and responding to subtle differences within various social and cultural spatial environments can optimize traffic circulation, improve infrastructure, and enhance safety measures, ultimately contributing to a better quality of life and a stronger sense of security (56).

For the satisfaction of higher-level needs (belonging and esteem needs), the physical spatial environment serves as a secondary yet structurally important supporting factor. Researchers suggest that high-quality built environments, exposure to natural elements, and well-designed public spaces contribute to improved psychological wellbeing, foster prosocial behaviors, enhance self-confidence, and strengthen social acceptance, which in turn indirectly facilitates the fulfillment of esteem needs (57–60).

The realization of self-actualization needs is significantly supported by the cultural spatial environment, particularly with respect to self-directed learning and personal growth. The diversity and accessibility of cultural resources and facilities strongly influence parents' willingness and likelihood to learn and develop new skills (61, 62). A well-developed community cultural environment facilitates the cultivation of interests and competencies beyond occupational and caregiving responsibilities, sustaining motivation for continuous self-improvement and developmental progression.

In summary, the development of a CCPSS requires the integrated consideration of both key and secondary environmental determinants to provide precise support for the multi-level needs of children and parents. The findings indicate that community environmental systems influence need satisfaction through differentiated mechanisms across hierarchical levels. Specifically, the physical spatial environment primarily secures basic physiological and safety needs by providing accessible and functional material conditions. Social and cultural spatial environments support belongingness and esteem needs by embedding individuals within shared social contexts and processes of recognition. Meanwhile, the social spatial environment and personalized behavioral environment contribute to self-actualization by generating sustained pathways for participation and development. Although secondary environmental factors are not the primary drivers of need fulfillment, they play an important facilitating and compensatory role in shaping individuals' environmental experiences and depth of engagement. In interaction with high-importance factors, they collectively structure the overall need-environment fit within the CCPSS.

This mechanism suggests that community childcare support is not a simple aggregation of single environmental elements. Rather, it operates through the coordinated functioning of multidimensional environmental systems across different levels of need, thereby providing systematic support for alleviating family parenting pressure and enhancing the wellbeing and overall quality of life of parenting groups.

### 5.3 Contributions and limitations

This study quantitatively analyzes the fit between the needs of children and parents and the community environment system, providing a more targeted and professional approach for optimizing the CCPSS. At the theoretical level, this study draws on P-E Fit Theory to develop an applied framework tailored to the community childcare and parenting context. By extending traditional single-subject fit approaches to a multi-actor setting in which children and parents coexist, the study broadens the application of P-E Fit Theory at the community scale and within the domain of childcare support, expanding its relevance to the interaction between needs and environmental systems.

At the analytical level, prior research on childcare-friendly environments has predominantly focused on basic needs, particularly physiological and safety requirements. In contrast, this study incorporates higher-order needs, such as belongingness, esteem, and self-actualization, thereby identifying differentiated pathways through which community environments contribute to the fulfillment of multi-level needs across childcare groups. This approach addresses the relative neglect of higher-order needs in existing community spatial research. Furthermore, this study explicitly incorporates parents' needs into the community spatial analysis framework, thereby addressing the limited consideration of parental perspectives in prior research and contributing to the development of a more comprehensive and coordinated CCPSS.

At the mechanistic level, this study demonstrates that community environments shape the multi-level need satisfaction of children and parents through tiered pathways, by providing foundational conditions that support childcare activities, embedding individuals within shared socio-cultural contexts, and generating sustained pathways for personal development. Through these mechanisms, community environments contribute to alleviating parenting-related pressures and enhancing the wellbeing of both children and parents, thereby providing empirical evidence to inform the renewal of parenting-friendly communities and the allocation of public services.

However, this study has several limitations. First, the sample size and research scope impose constraints on the generalizability of the findings. The study was conducted exclusively in Heping District, Tianjin, with a relatively limited sample, making it more reflective of the specific socio-environmental conditions of this region rather than a universally applicable model. Future research should expand to different urban and rural contexts to account for regional variations in CCPSS implementation. Second, while this study employed a questionnaire survey-based data collection, which is widely used in social science research, it has inherent limitations. Future studies could integrate multi-source big data analytics, such as social media data mining, web scraping, and spatial data analysis, to improve data accuracy, depth, and dynamic representation.

In conclusion, this study provides theoretical and empirical insights for the construction of parenting friendly communities, offering a scientific foundation for future urban planning and policy development. Future studies should focus on broadening sample diversity, refining need analysis, and optimizing data collection methodologies to establish a more comprehensive, systematic, and sustainable CCPSS.

## 6 Conclusion

This study examines the fit between childcare and parenting needs and community environments within the CCPSS framework through Pearson correlation analysis and random forest modeling, applying P-E Fit Theory to construct a refined, evidence-based support framework. Unlike prior research that emphasizes basic physiological and safety needs, this study integrates hierarchical need fulfillment, extending the scope of CCPSS to include social belonging, esteem, and self-actualization needs. The results indicate significant positive correlations between the satisfaction of children's and parents' needs and different environmental systems, emphasizing that physical spatial environments are the most critical for meeting basic physiological and safety needs, while cultural and social environments play a pivotal role in fostering belonging, esteem, and personal growth.

Additionally, secondary environmental factors, though not primary drivers, significantly influence individual experiences and interactions within childcare and parenting supportive communities. These findings highlight the importance of integrated community planning that aligns infrastructure, cultural resources, social networks and support service to enhance child-friendly and parenting-supportive environments.

Future research should expand to diverse urban and rural settings and incorporate multi-source data analytics to improve the scalability and adaptability of the CCPSS model. These insights provide a scientific basis for policymakers and urban planners, facilitating more targeted, need-responsive childcare and parenting policies and community designs.

## Data Availability

The original contributions presented in the study are included in the article/supplementary material, further inquiries can be directed to the corresponding author.
